# Supplementary material for: A UMLS-based spell checker for natural language processing in vaccine safety
Source: BMC Med Inform Decis Mak. 2007 Feb 12;7:3. doi: 10.1186/1472-6947-7-3 (PMC1805499; doi:10.1186/1472-6947-7-3)
Supplement: Additional file 13 — RAP application directory. Sets up directory for RAP (RDF application for PHP) source code [file 1472-6947-7-3-S13.gz › rap/doc/phpdoc/model/Statement.html]

xml version="1.0" encoding="iso-8859-1"?


Docs For Class Statement


## Class Statement

Description

Description |
Descendents
| Methods (details)

An RDF statement.

In this implementation, a statement is not itself a resource. If you want to use a a statement as subject or object of other statements, you have to reify it first.

History:

- version: V0.9.1
- author: Chris Bizer <chris@bizer.de>

Located in /model/Statement.php (line 22)

```
Object
   |
   --Statement
```

Direct descendents

Description |
Descendents
| Methods (details)

| Class | Description |
| --- | --- |
| InfStatement | An RDF statement which was entailed by a inference rule. |

Method Summary

Description |
Descendents |
Methods (details)

Statement
Statement
(object node $subj, object node $pred, object node $obj)

boolean
compare
(object statement &$that)

boolean
equals
(object statement $that)

string
getLabelObject
()

string
getLabelPredicate
()

string
getLabelSubject
()

object node
getObject
()

object node
getPredicate
()

object node
getSubject
()

string
hashCode
()

object node
object
()

object node
predicate
()

object model
&reify
(mixed &$model\_or\_bNodeID)

object node
subject
()

string
toString
()

string
toStringObject
()

string
toStringPredicate
()

string
toStringSubject
()

Methods

Description |
Descendents |
Methods (details)

Constructor Statement (line 56)

The parameters to constructor are instances of classes and not just strings

- throws: PhpError

Statement

Statement
(object node $subj, object node $pred, object node $obj)

- object node
  $subj
- object node
  $pred
- object node
  $obj

compare (line 246)

Compares two statements and returns integer less than, equal to, or greater than zero.

Can be used for writing sorting function for models or with the PHP function usort().

- access: public

boolean

compare
(object statement &$that)

- object statement
  &$that

equals (line 222)

Checks if two statements are equal.

Two statements are considered to be equal if they have the same subject, predicate and object. A statement can only be equal to another statement object.

- access: public

boolean

equals
(object statement $that)

- object statement
  $that

getLabelObject (line 208)

Reurns the URI, text or bNode identifier of the statements's object.

- access: public

string

getLabelObject
()

getLabelPredicate (line 198)

Returns the URI of the statements's predicate.

- access: public

string

getLabelPredicate
()

getLabelSubject (line 188)

Returns the URI or bNode identifier of the statements's subject.

- access: public

string

getLabelSubject
()

getObject (line 102)

Returns the object of the triple.

- access: public

object node

getObject
()

getPredicate (line 93)

Returns the predicate of the triple.

- access: public

object node

getPredicate
()

getSubject (line 84)

Returns the subject of the triple.

- access: public

object node

getSubject
()

hashCode (line 137)

Retruns the hash code of the triple.

- access: public

string

hashCode
()

object (line 129)

Alias for getObject()

- access: public

object node

object
()

predicate (line 120)

Alias for getPredicate()

- access: public

object node

predicate
()

reify (line 262)

Reifies a statement.

Returns a new MemModel that is the reification of the statement. For naming the statement's bNode a Model or bNodeID must be passed to the method.

- access: public

object model

&reify
(mixed &$model\_or\_bNodeID)

- mixed
  &$model\_or\_bNodeID

subject (line 111)

Alias for getSubject()

- access: public

object node

subject
()

toString (line 147)

Dumps the triple.

- access: public

string

toString
()

---

Redefinition of:

Object::toString()
:   Serializes a object into a string

toStringObject (line 178)

Reurns a toString() serialization of the statements's object.

- access: public

string

toStringObject
()

toStringPredicate (line 168)

Returns a toString() serialization of the statements's predicate.

- access: public

string

toStringPredicate
()

toStringSubject (line 158)

Returns a toString() serialization of the statements's subject.

- access: public

string

toStringSubject
()

#### Inherited Methods


Inherited From Object

> Object::toString()

Documentation generated on Thu, 7 Jul 2005 13:43:56 +0200 by phpDocumentor 1.3.0RC3
